# Immobilization of Titanium(IV) Oxide onto 3D Spongin Scaffolds of Marine Sponge Origin According to Extreme Biomimetics Principles for Removal of C.I. Basic Blue 9

**DOI:** 10.3390/biomimetics2020004

**Published:** 2017-03-25

**Authors:** Tomasz Szatkowski, Katarzyna Siwińska-Stefańska, Marcin Wysokowski, Allison L. Stelling, Yvonne Joseph, Hermann Ehrlich, Teofil Jesionowski

**Affiliations:** 1 Institute of Chemical Technology and Engineering, Faculty of Chemical Technology, Poznan University of Technology, Berdychowo 4, Pl-60965 Poznan, Poland; tomasz.p.szatkowski@doctorate.put.poznan.pl (T.S.); katarzyna.siwinska-stefanska@put.poznan.pl (K.S.-S.); marcin.wysokowski@put.poznan.pl (M.W.); 2 Department of Biochemistry, Duke University, 307 Research Drive, Durham, NC 27710, USA; Allison.Stelling@duke.edu; 3 Institute of Electronics and Sensor Materials, TU Bergakademie Freiberg, Gustav-Zeuner-Str. 3, 09599 Freiberg, Germany; yvonne.joseph@esm.tu-freiberg.de; 4 Institute of Experimental Physics, TU Bergakademie Freiberg, Leipziger Str. 23, 09599 Freiberg, Germany

**Keywords:** extreme biomimetics, spongin, scaffolds, marine sponges, hydrothermal synthesis, titanium dioxide, photocatalysis

## Abstract

The aim of extreme biomimetics is to design a bridge between extreme biomineralization and bioinspired materials chemistry, where the basic principle is to exploit chemically and thermally stable, renewable biopolymers for the development of the next generation of biologically inspired advanced and functional composite materials. This study reports for the first time the use of proteinaceous spongin-based scaffolds isolated from marine demosponge *Hippospongia communis* as a three-dimensional (3D) template for the hydrothermal deposition of crystalline titanium dioxide. Scanning electron microscopy (SEM) assisted with energy dispersive X-ray spectroscopy (EDS) mapping, low temperature nitrogen sorption, thermogravimetric (TG) analysis, X-ray diffraction spectroscopy (XRD), and attenuated total reflectance–Fourier transform infrared (ATR–FTIR) spectroscopy are used as characterization techniques. It was found that, after hydrothermal treatment crystalline titania in anatase form is obtained, which forms a coating around spongin microfibers through interaction with negatively charged functional groups of the structural protein as well as via hydrogen bonding. The material was tested as a potential heterogeneous photocatalyst for removal of C.I. Basic Blue 9 dye under UV irradiation. The obtained 3D composite material shows a high efficiency of dye removal through both adsorption and photocatalysis.

## 1. Introduction

Incorporation of inorganic compounds and biological macromolecules using a nature-inspired biomimetic approach can provide remarkably attractive solutions to current technological challenges, and lead to the development of novel, advanced composite materials that exhibit unique physicochemical, structural, and functional properties [1,2,3,4]. The highly interdisciplinary field of biomimetics involves the understanding of biological functions, structures, and principles of various objects found in nature by scientists from diverse disciplines. From the commercial point of view, examples of inspiring natural solutions include such phenomena as superhydrophobicity, self-cleaning, drag reduction in fluid flow, energy conversion and conservation, high adhesion, aerodynamic lift, materials and fibers with high mechanical strength, biological self-assembly, structural coloration, thermal insulation, and self-healing (for a review, see [5]).

Recently, a novel interdisciplinary scientific field, termed “extreme biomimetics,” has emerged [6,7]. From the fundamental science point of view, it aims at understanding of the principles of extreme biomineralization reactions occurring in the extreme niches of the Earth, such as hydrothermal (deep-sea vents, geothermal pipelines) or psychrophilic (freezing Antarctic and Arctic waters) environments. From a practical science point of view, it opens the door to the development of novel composite materials using both thermostable biopolymers (chitin, spongin, silk) and selected inorganic phases under hydrothermal synthesis conditions [8,9,10]. This direction has gained enormous attention especially in aspects where there is an interest in the development of materials, which feature not only hierarchical and nanostructural organization but three-dimensional (3D) architecture at micro- and macrolevels. Such kind of 3D scaffolds can be isolated from diverse marine demosponges, which are cultivated worldwide under marine ranching conditions [11]. Mostly, their fibrous skeletons are made of aminopolysaccharide chitin (sponges in the order Verongida) or proteinaceous spongin (representatives of the order Dictyoceratida).

Biological templates with 3D morphology, such as those seen in chitin-based sponges in the order Verongida [12,13,14,15], are thermostable up to 400 °C, and consequently have already been used for the development of metal oxide-containing composites in the form of scaffolds, with specific properties under hydrothermal conditions [6,16,17]. For example, chitin isolated from Verongida sponges was used as a natural matrix for the deposition of zirconia [6,16], zinc oxide [17], hematite [10,18], and germanium dioxide [9] composites.

Spongin also possesses interesting properties with respect to thermostability between 150 °C and 360 °C [19]. This collagen- and keratin-like structural protein represents the main basic element of keratosan demosponges skeletons, which are characterized as fibrous, mechanically rigid frameworks with a high porosity very useful for tissue engineering and tissue bionics [20,21,22,23]. In order to provide a skeletal support to the bulk of sponge cell tissue, spongin is arranged in an anastomosed open-pores network of horny fibers, which are highly efficient for water filtration necessary for food collection. Amazingly, the chemical and thermal performance of spongin is very comparable to keratin. It is highly resistant to mild acidic or basic hydrolysis. Enzymatic treatment using collagenase, pepsin, trypsin, chymotrypsin, pronase, papain, elastase, lysozome, cellulase, or α-amylase hardly affect the structure of spongin as well [19]. What are the perspectives for the use of spongin as a renewable and naturally prestructured 3D scaffold in the direction of extreme biomimetics, which is oriented towards hydrothermal synthesis reactions?

Implementation of the extreme biomimetic approach in a laboratory can be relatively easy with the use of hydrothermal synthesis techniques. Hydrothermal processes are carried out in aqueous solvents or mineralizers under high pressure and temperature conditions to dissolve and recrystallize (recover) materials that might be relatively insoluble under ordinary conditions [24]. This technique also allows for control of the reaction to a high extent; thus, cleaner products can be obtained in a more efficient and often “greener” manner. Moreover, hydrothermal techniques allow for the creation of unique structures that are hard or impossible to obtain using low temperature methods.

In our previous study, we synthesized hematite nanoparticles in the presence of spongin fibers originating from the *Hippospongia communis* demosponge [25]. The results obtained in this study motivated us to carry out experiments with other metal oxides such as titanium oxide. A study of the synthesis of titania in the presence of spongin-based scaffolds has not been reported before, but the immobilization of titania nanoparticles onto alternative proteinaceous substrates (e.g., wool or feather keratin) has been extensively studied [26,27,28].

The idea of an incorporation of TiO_2_ into fibrous materials is a new concept, although the attractive features of the material as a photocatalyst are widely recognized. The fact that TiO_2_ is extensively used in that field also results from its non-toxicity, and both physicochemical and thermal stability. Due to a series of reduction and oxidation reactions occurring during photocatalysis under UV light, titania nanoparticles have been found to be useful in the removal of water pollutants [29,30,31] and air contaminants [32,33,34], surface self-cleaning [35,36,37], antimicrobial applications [37,38,39], and UV protection [40,41,42]. In addition to its photocatalytic activity, TiO_2_ is used as powerful catalyst support due to its possible strong metal support interaction (SMSI) [43] and its feasibility of enabling spill-over effects [44].

Despite the catalytic applications, porous TiO_2_ is also used as a charge transfer layer in dye-sensitized solar cells (DSSCs) [45]. Here, the morphology and the nature of the surface of the TiO_2_ network is extremely important for the efficiency of the solar cell [46]. Several scientists have created heterogeneous systems using various substrates for the immobilization of titania, as reviewed by Shan et al. [47], which, although have diminished surface availability, do eliminate the need for filtration. Still, there are no reports on the use of 3D spongin-containing scaffolds of marine demosponge origin as a support for hydrothermally obtained titanium(IV) oxide. The hydrothermal synthesis of titania results in the crystalline form of anatase, which is considered as the most efficient in the photocatalysis processes [48]. Moreover, the high temperature treatment, which follows the principles of extreme biomimetics, is a common method to anchor the titanium dioxide photocatalyst to its support [49].

## 2. Materials and Methods

### 2.1. Isolation of Spongin Scaffolds

**Figure 1:**
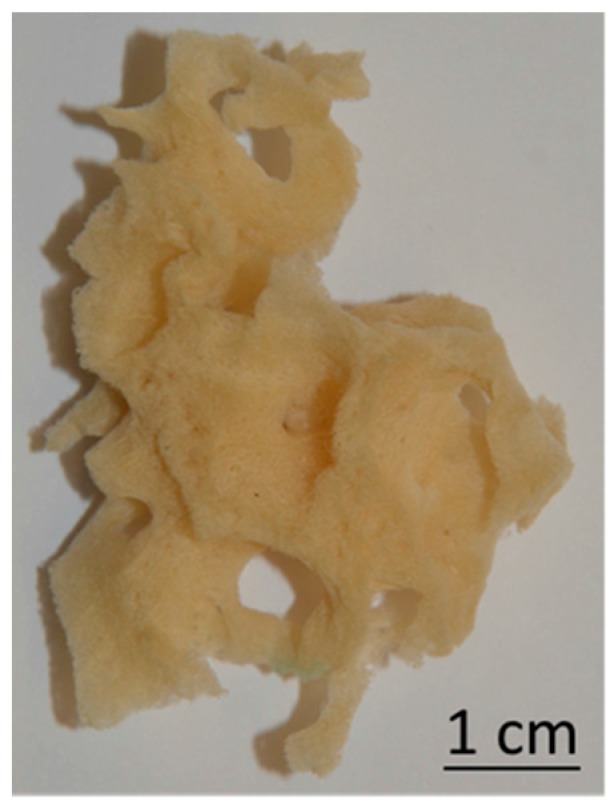
Mineral- and pigment-free three-dimensional spongin scaffolds isolated from *Hippospongia communis* demosponge.

The specimens of marine demosponges *Hippospongia communis* (Porifera, Demospongiae, Keratosa, Dictyoceratida, Spongiidae, *Hippospongia*) were collected from the Mediterranean Sea at Tunisia’s coast, and isolated spongin scaffolds were directly provided to the laboratory by BromMarin (Freiberg, Germany). Prior to use, the sponge fragments were washed again with running water in order to remove any major contamination. Afterwards, the spongin skeleton was purified with use of 3.0 M hydrochloric acid provided by Sigma-Aldrich (Munich, Germany) at room temperature to remove natural calcium carbonate-containing impurities. After 72 h, the scaffolds were washed with distilled water until the pH of eluted water was equal to about 6–7. Washed scaffolds (Figure 1) were dried at 50 °C for 24 h and stored at room temperature in plastic bags.

### 2.2. Hydrothermal Synthesis of TiO_2_ Immobilized onto Spongin Scaffold

The pre-treated fragments of selected spongin scaffolds were soaked in 30 cm^3^ of precursor solution, i.e., titanium(IV) butoxide (TBOT) (Sigma-Aldrich), for 24 h at room temperature and stored in a desiccator to prevent reaction with air humidity. After that time, the scaffolds with TBOT were transferred into the hydrothermal reactor (Hydrion-Scientific, Baltimore, MD, USA) and stored at 120 °C for 3 h. To remove all unbound particles of titanium dioxide, the TiO_2_ immobilized onto spongin (SpI–TiO_2_) was cleaned using an ultrasound bath (Elmasonic GmbH, Singen, Germany) for 1 h and dried at 50 °C for 24 h.

The TiO_2_ used in measurements as a reference sample was synthesized in analogous way as the spongin-based composite using hydrothermal conditions (120 °C for 3 h), but without the presence of skeleton of *H. communis*. After synthesis, the powdered titania was dried at 100 °C for 24 h.

### 2.3. Characterization Techniques

Scanning electron microscopy (SEM) images were recorded using EVO40 scanning electron microscope (Zeiss, Munich, Germany) equipped with an energy dispersive X-ray spectroscooopy (EDS) detector (Princeton Gamma-Tech Instruments Inc., Princeton, NJ, USA). Prior to testing, the samples were coated with Au over a period of 15 s using a Balzers PV205P coater (Brügg, Switzerland).

Low-temperature nitrogen sorption (−196 °C) was applied for the evaluation of parameters of the porous structure. An ASAP 2020 surface analyzer (Micromeritics Instrument Co., Norcross, GA, USA) was used. Before the analysis, the obtained samples were degassed in a vacuum at 120 °C for 4 h. The surface area was calculated according to the Brunauer–Emmett–Teller (BET) method, and the pore size distribution was determined by means of the Barrett–Joyner–Halenda algorithm.

Thermogravimetric (TG) analysis of spongin and SpI–TiO_2_ material was performed via use of a Jupiter STA 449F3 (Netzsch, Selb, Germany) analyzer. The samples were placed in an Al_2_O_3_ crucible and measured in an air atmosphere at a heating rate of 10 °C·min^−1^ in a temperature range of 25 °C to 1000 °C.

X-ray diffraction (XRD) analyses were performed using a TUR-M62 diffractometer (Freiberger Präzisionsmechanik Holding GmbH, Freiberg, Germany), operating at 30 kV and 25 mA, with CuKα (a = 1.5418 Å) radiation, Ni filtered. The XRD pattern data were collected in step-scanning mode with steps of Δ2θ = 0.04°, over an angular range of 5°–60°.

The infrared spectroscopy was performed with a Vertex 70 spectrometer (Bruker Optics, Ettlingen, Germany) equipped with a Platinum ATR (attenuated total reflectance) accessory (Bruker Optics, Ettlingen, Germany), using a single reflection diamond crystal, in the range of mid-infrared ATR (MIR-ATR). The powdered sample underwent the measurement under pressure of the clamp mechanism. This technique can be utilized directly in the solid or liquid state and does not destroy the protein structure of the sample, contrary to standard Fourier transform infrared (FTIR) spectroscopic methods, which demands the preparation of samples in the form of a KBr pellet.

The photocatalytic activity of the obtained samples was evaluated by the degradation of model organic impurity—an aqueous solution of C.I. Basic Blue 9 dye (Sigma-Aldrich) in an initial concentration of 5 mg/L under UV irradiation. The model organic impurity and the photocatalyst were placed in a laboratory reactor of UV-RS2 type (Heraeus, Hanau, Germany), equipped with a 150 W medium-pressure mercury lamp as a UV light source. Before UV irradiation, the obtained suspension was magnetically stirred in darkness for 30 min to ensure that adsorption/desorption equilibrium of the reaction solution was attained. After this time, the radiation was turned on to initiate the photocatalytic reaction. The process was conducted for a time of 60 min. The irradiated mixtures were separated by filtration, and the concentrations of C.I. Basic Blue 9 in an aqueous solution, before and after UV irradiation, were monitored by measuring light absorption at 664 nm with a SPEKOL UV-1201 spectrophotometer (Shimadzu, Kyoto, Japan), using water as a reference.

## 3. Results and Discussion

**Figure 2:**
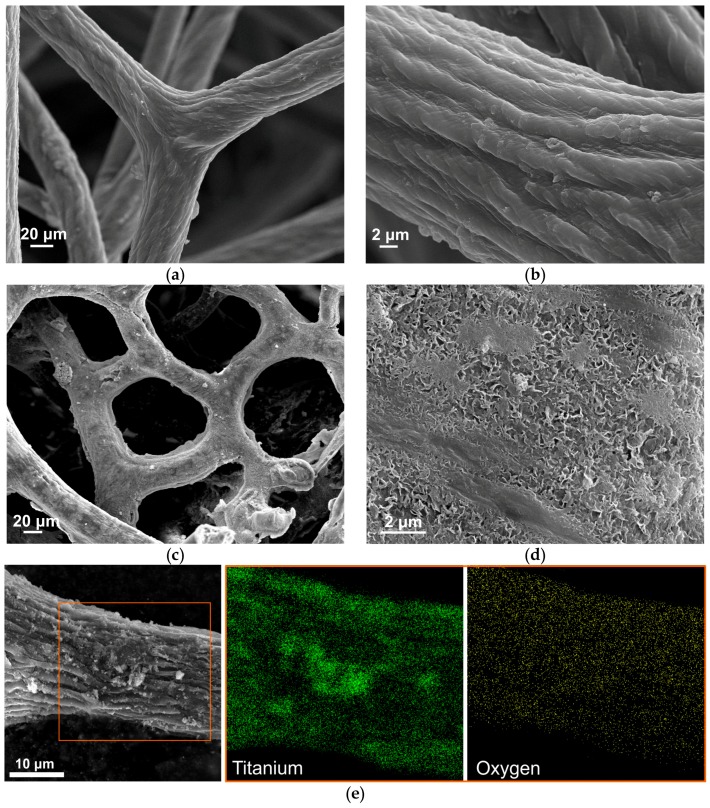
Scanning electron microscopy (SEM) images. (**a**,**b**) Spongin fibers before the hydrothermal treatment in titanium(IV) butoxide (TBOT); (**c**,**d**) TiO_2_ immobilized onto spongin (SpI–TiO_2_) obtained through hydrothermal synthesis; (**e**) local energy dispersive X-ray spectroscopy (EDS) measurements. The structure remains stable even after 1 h of ultrasonic treatment.

SEM images presented in Figure 2 show the surface morphology of spongin fibers before and after hydrothermal synthesis of titanium dioxide at a temperature of 120 °C. In Figure 2a,b, one can observe a network of spongin microfibers creating complex, foam-like formations. The 3D structure of *H. communis* skeletons ensures remarkable mechanical performance, useful primarily against strong water currents and predation. On the other hand, it is easy to notice the open porosity of the skeleton, which is crucial for the efficient collection of food through a filtration of surrounding waters.

At a closer glance, microfibrils comprising the fibers in an intertwisted manner are noticeable, and create a Bouligand structure [50]. SEM images in Figure 2c,d show the spongin fiber almost completely covered by titania, which is exclusively deposited on the surface of the fibers. Formation of the TiO_2_ inside the fibers is impossible due to the lack of a hollow interior in the spongin. This high quality of the inorganic coating might be owed to the synthesis procedure (a penetration of the spongin scaffold by the TBOT for 24 h) as well as to hydrothermal treatment. Importantly, the unique 3D structure of the sponge skeleton is conserved even after high-temperature treatment. At higher magnification (Figure 2d), it is possible to observe a detailed surface structure of the titanium dioxide coating formed around the spongin fibers. In Figure 2e, the results of EDS analysis are presented, which was carried out by elemental mapping, showing that the single fiber is homogeneously covered by titania.

**Figure 3:**
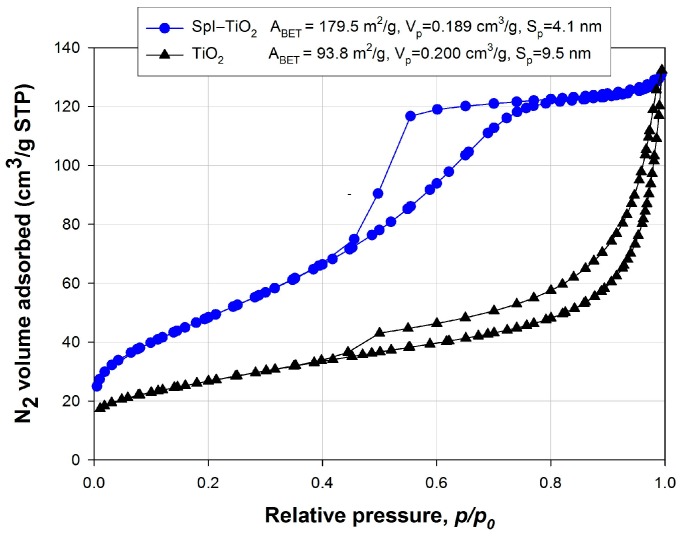
N_2_ adsorption/desorption isotherms of obtained materials. A_BET_: Specific surface area; S_p_: Mean pore diameter; V_p_: Total pore volume; STP: Standard temperature and pressure.

One of the parameters that determine the photocatalytic activity of obtained materials is the porous structure. Figure 3 presents low-temperature N_2_ sorption isotherms for TiO_2_ and SpI–TiO_2_ material. The adsorption/desorption isotherm measured for the reference TiO_2_ sample was classified as a type II with a hysteresis loop type H4, indicating the mesoporous nature of the material. The BET surface area of titanium dioxide is 93.8 m^2^/g, the total pore volume of this sample is 0.200 cm^3^/g, and the mean pore diameter is 9.5 nm. The hysteresis loop of synthetic titania covers the relative pressure range *p/p_0_* = 0.40–0.99. The amount of nitrogen adsorbed slowly increases in the relative pressure range *p/p_0_* = 0.0–0.8; above *p/p_0_* = 0.8, it rapidly increases to reach a maximum value of 130 cm^3^/g at *p/p_0_* = 0.99. Significantly different characteristics of the porous structure were observed for the SpI–TiO_2_ hybrid material. The isotherm for the mentioned sample is classified as type IVA with the hysteresis loops H2. The obtained hybrid material is characterized by a surface area of 179.5 m^2^/g, higher in comparison to the reference TiO_2_. The hysteresis loop of this sample covers the relative pressure range of *p/p_0_* = 0.4–0.8. Additionally, SpI–TiO_2_ material is characterized with a total pore volume of 0.189 cm^3^/g and a pore diameter of 4.1 nm.

The synthesis of SpI–TiO_2_ hybrid material led to the obtained product characterized by better parameters of the porous structure.

**Figure 4:**
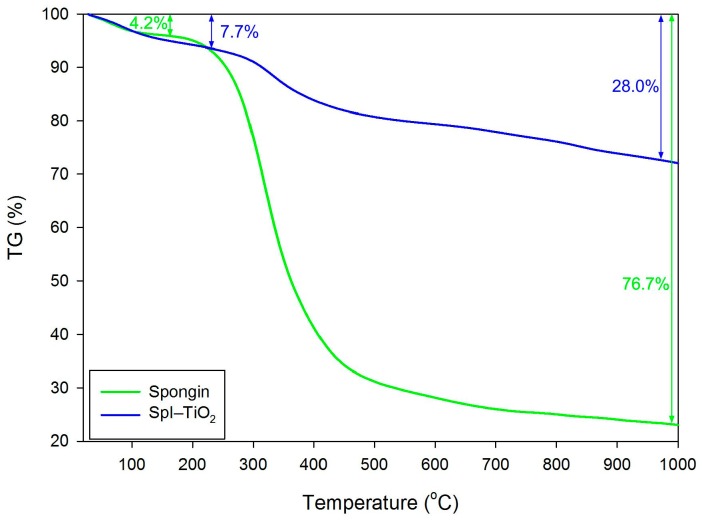
Thermogravimetric (TG) curves of spongin and SpI–TiO_2_ composite, showing the mass loss of samples during heating.

In order to estimate the amount of TiO_2_ deposited on the surface, thermogravimetric analysis has been performed (Figure 4). First mass change occurring at a temperature of up to 140 °C is ascribed to the evaporation of water molecules. It can be noticed that more water was bound to the SpI–TiO_2_ composite (equal to 7.7%), which is owed to the larger surface area of the material. Further mass loss characterized with a narrow drop of the curves originates from the destruction of protein structure of the sponge skeleton. For pure spongin, the mass loss is equal to as much as 76.7%, while for the composite it equals 28.0%. The remnants of spongin heated to 1000 °C account for 23.3% of the whole material, which indicates that the SpI–TiO_2_ contains about 48.7% of TiO_2_ coating.

**Figure 5:**
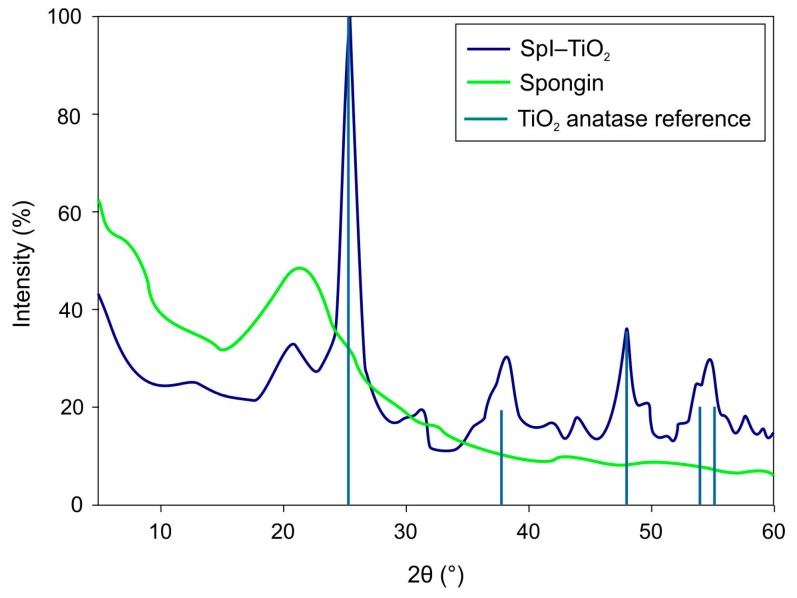
X-ray diffraction (XRD) patterns of SpI–TiO_2_ composite accompanied by reference peaks characteristic of anatase and compared with a pattern recorded for purified spongin scaffold.

The XRD patterns of the obtained 3D composite, which allow for the identification of the titania coating, are presented in Figure 5. The pattern measured for SpI–TiO_2_ shows four major peaks pointing at the characteristic crystallographic phase of anatase, which are well-aligned with the reference bars of anatase according to the Joint Committee on Powder Diffraction Standards (JCPDS) database (Card No. 21-1272). The main peak of the highest intensity appears at a 2θ angle of about 25.4° and is related to *hkl* index (101). The three remaining peaks present at the 2θ angles 38.1°, 48.8°, and 54.5° are ascribed to *hkl* indexes (004), (200), and (211), respectively, which is in agreement with literature data [51]. The pattern recorded for spongin does not show any sharp peaks, but the curve’s profile is relatively similar to the one recorded for keratin fibers, indicating their analogical structure [52]. Importantly, in the pattern of the SpI–TiO_2_ material, at 2θ angles of 20°, a low intensity peak occurs originating from spongin.

In order to understand the nature of interactions between the proteinaceous matrix of spongin and TiO_2_ coating, it is important to take a closer look into the chemistry of the biomaterial [7]. Although the first experiments on the skeleton of demosponges (known as common bath sponges) were conducted as early as the beginning of the 19th century, the amount of available literature data is relatively limited. Nonetheless, it is known that spongin is built of amino acids in a manner similar to the collagen, which is comprised according to a Gly–X–Y triplet motif, where the X- or Y-position is usually occupied by hydroxyproline and the remaining position by any of the present amino acids [53]. Hydroxyproline, which often serves as a diagnostic tool of collagen in any organism, was found in high amounts in several sponges of Dictyoceratida (Demospongiae) [54]. Similar collagen concentrations in other amino acids, such as glycine, tyrosine, arginine, and histidine, are typical [22,54,55,56]. Amino acids present in the structure of spongin are a source of functional groups that play an important role in the interaction with other compounds. The concentration of amino acids in *H. communis* marine sponge has been estimated by Junqua et al. [19]. According to the report, spongin is composed of about 19 different amino acids, among which the highest concentration (percentage content per 100 g of spongin) was identified for glycine (31.9%), aspartic acid (9.4%), hydroxyproline (8.7%), alanine (8.4%), and glutamic acid (7.9%). These protein building blocks are characterized with the presence of functional groups including –COOH, –NH_2_, and –OH, which are responsible for effective interactions with other substances such as TiO_2_.

**Figure 6:**
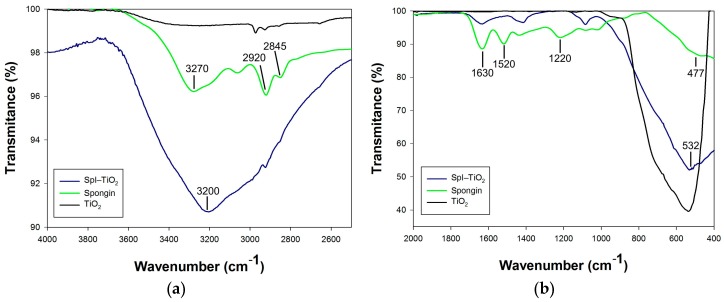
The attenuated total reflectance–Fourier transform infrared (ATR–FTIR) spectra of spongin, titania (reference sample, anatase), and SpI–TiO_2_ composite obtained via hydrothermal synthesis, presented as spectra in two ranges: (**a**) in wavenumber range 4000–2500 cm^−1^, and (**b**) in range of 2000–400 cm^−1^.

In Figure 6, the attenuated total reflectance–Fourier transform infrared (ATR–FTIR) spectra of spongin, titania, and the SpI–TiO_2_ composite are presented, showing some important information regarding their chemical properties and the presence of functional groups. The broad peak in the range of 3200–3300 cm^−1^ at the spongin spectrum originates from the N–H stretching vibration comprising the amide A band, and the O–H stretching vibrations (Figure 6a). Two peaks with maxima at 2920 cm^−1^ and 2845 cm^−1^ are related to CH_3_ and CH_2_ symmetric and asymmetric vibrations, respectively. In the spectrum of SpI–TiO_2_, those three important peaks are overlapping to a high extent with broad peak characteristics for –OH groups, indicating the presence of hydrogen-bonded water molecules (H–O–H···H) and plausible titanium-bonded hydroxyl groups. In Figure 6b, at 1630 cm^−1^ and 1520 cm^−1^, CO and N–H stretching of amide I (mainly CO stretching vibration) and Amide II (N–H bending and C–H stretching vibration) produce relatively intensive peaks, which are overlapping with the H–O–H water bending vibration (typically at 1640 cm^−1^). The intensity of these peaks is diminished in the spectrum of SpI–TiO_2_. Moreover, the Amide III band occurring in the range of 1220–1300 cm^−1^ in the spectrum of spongin—resulting from the in phase combination of C–N stretching and N–H plane bending vibrations, which contributes to the footprint of peptide bonds (–CONH–) [57]—is significantly reduced in the spectrum recorded for the organic–inorganic material. Finally, at lower wavenumbers, one can observe the N–H band with a maximum at 477 cm^−1^, which is overlapping with a high-intensity Ti–O band at 532 cm^−1^ in the spectrum of the SpI–TiO_2_, which is also clearly observable in the spectrum of reference TiO_2_, which has a crystalline form of anatase and was prepared in analogical conditions as the examined composite.

The functional groups on spongin surfaces that can be considered potential reaction sites for binding with TiO_2_ particles are the carboxyl (–COOH), amino (–NH_2_), and hydroxyl (–OH) groups. The high affinity of titanium dioxide particles to negatively charged hydroxyl and carboxyl groups has already been studied [58]. Based on the observation of ATR–FTIR spectra of spongin before and after the hydrothermal synthesis of titania, it can be deduced that TiO_2_ particles exhibit stronger affinity to functional groups of spongin without any need for surface modification. The interaction between TiO_2_ and the spongin network can be considered both chemical (e.g., condensation mechanism, electrostatic interactions) and physical bonding (e.g., hydrogen interactions).

**Figure 7:**
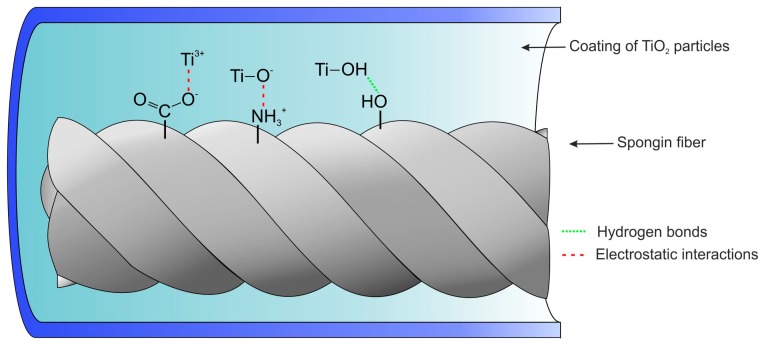
Proposed mechanism of titanium dioxide immobilization onto spongin fibers involving hydrogen bonding and electrostatic interactions.

Based on the obtained data and literature reports regarding TiO_2_–protein interactions [59,60,61,62,63], we proposed a plausible mechanism of titanium dioxide bonding to the surface of spongin (see Figure 7). Multiple functional groups are present at the surface of spongin and might be binding positions involved in the bonding of titanium dioxide. However, carbonyl and amine groups are known to play a particularly important role in the bonding between protein and metal oxide. It has been confirmed that the negatively charged oxygen atom of carbonyl (–COO^−^) and the positive charge located at the amine group (–NH^3+^) are responsible for electrostatic interaction between the two materials [64]. What is more, several studies have confirmed that hydrothermal treatment positively influences the amount of adsorbed TiO_2_ particles onto the surface of protein [27]. The presence of hydroxyl groups at the surface of titania, as indicated by the ATR–FTIR spectra, also strongly contributes to the strength of adsorption between the two materials through hydrogen bonding. It is also known that high-temperature treatment might generate titanium trivalent (Ti^3+^) on the surface of TiO_2_ particles, which is very reactive [64,65] and plays a crucial role in the photocatalytic process. Thus, it was also considered in the proposed mechanism of immobilization of titanium dioxide onto spongin fibers.

Finally, the SpI–TiO_2_ composite with 3D architecture was tested as a heterogeneous photocatalyst for the removal of C.I. Basic Blue 9 dye from an aqueous solution. The mechanism behind the photocatalysis is well known and can be summarized in the following manner: TiO_2_ produces excited high-energy states of electron and hole pairs (e^−^/h^+^) due to the illumination with light energy (*hν*) greater than the bandgap energy (*E_g_*) of the semiconductor, which for TiO_2_ equals 3.2 eV (*hν* > *E_g_*). The photogenerated carriers partly recombine in the bulk of the titania, but some migrate to the surface of particles, where the holes and electrons act as powerful oxidants and reductants, respectively. They initiate a wide range of redox reactions, eventually leading to the mineralization of dye through nonselective attack of the adsorbed molecules [66,67,68]. The photodegradation process can be expressed through the following equations:

(1) $${\mathrm{TiO}}_{2}+h\nu \to e^{-}+h^{+}$$

(2) $$O_{2\mathrm{ads}}+e^{-}\to O^{•-}{}_{\mathrm{ads}}$$

(3) $$h^{+}+H_{2}O_{\mathrm{ads}}\to {\mathrm{HO}}^{•}+H^{+}$$

(4) $$h^{+}+{\mathrm{HO}}^{-}{}_{\mathrm{ads}}\to {\mathrm{HO}}^{•}$$

(5) $$R-H+{\mathrm{HO}}^{•}\to {\mathrm{RCOO}}^{•}\to {\mathrm{CO}}_{2}+H_{2}O+\mathrm{inorganic}\;\mathrm{ions}.$$

**Figure 8:**
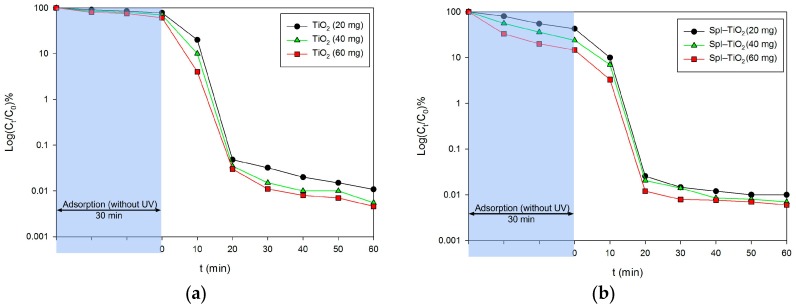
Photodegradation efficiency of C.I. Basic Blue 9 using (**a**) TiO_2_ particles dispersed in the solution and (**b**) TiO_2_ immobilized on spongin scaffolds presented as a function of quotient of dye concentration at time t (C_t_) and the initial dye concentration (C_0_) versus irradiation time.

The photocatalytic degradation of the C.I. Basic Blue 9 in the presence of dispersed titanium dioxide (obtained via hydrothermal technique in analogical conditions as the examined composite) as well as TiO_2_ immobilized onto spongin scaffolds is presented in Figure 8. The measurement was carried out for various masses of materials, equal to 20 mg, 40 mg, and 60 mg. The graphs involve the initial 30 min period of stirring without UV irradiation, presenting the amount of dye adsorbed on the photocatalyst particles. As can be clearly noticed, the amount of adsorbed dye is significantly higher when the support of spongin scaffolds is used (as much as 85.4% of dye is adsorbed when 60 mg of the sample is used), while only 25.0% of the dye is adsorbed in the case of 60 mg of titania dispersion. These observations correlate well with the difference between the two materials in terms of specific surface area (Figure 3). The majority of model contamination is photocatalyzed after only 20 min, and the curve starts reaching its equilibrium after that period of time. After 60 min of the process, a near-complete removal of dye can be observed in the case of dispersed TiO_2_, but titania immobilized onto the skeleton of marine sponge is more efficient and removes the dye after only 30 min. Blank experiments were carried out by irradiating the model aqueous solution of the dye in the absence of TiO_2_, showing that, after 60 min of irradiation, only 27.7% loss of the compound was observed. Similar experiments were carried out by Zhang et al., who tested TiO_2_-coated wool fibers using the same concentration of C.I. Basic Blue 9 dye [26]. The photocatalytic study showed that more than 95% of the dye was degraded after 180 min of UV irradiation, although it has to be kept in mind that the power of UV lamp was smaller. The development of a new generation of functional materials with photocatalytic and UV-barrier properties based on nano-TiO_2_ and textile support was previously described by Siwińska-Stefańska et al. [69]. Moreover, commercial titania modified with selected alkoxysilanes introduced into the structure of polyester fabric proved the enhanced photooxidative activity with the photodegradation of formaldehyde under UV radiation [70].

## 4. Conclusions and Future Perspectives

For the first time, a TiO_2_ coating has been synthesized using a hydrothermal mineralization route onto 3D spongin-based scaffolds isolated from *H. communis* marine demosponge as a porous support according to an extreme biomimetics approach. The SEM images assisted with EDS mapping show that the coating is uniformly and firmly attached to the surface of the fibers, and is not removed even after ultrasonic treatment. It can be observed that the proteinaceous matrix strongly increases the surface area, which was depicted by the nitrogen sorption isotherms. The strong adherence of inorganic nanoparticles to the proteinaceous support might result from the presence of functional groups originating from the amino acid composition of the material. The XRD pattern shows that the high-temperature treatment allowed the titania to be obtained in a crystalline form of anatase, which is considered highly efficient in photocatalysis processes. The titanium dioxide particles immobilized onto spongin fibers proved itself efficient in the removal of C.I. Basic Blue 9 through both adsorption and photocatalysis.

These highly porous, stable 3D spongin-based scaffolds coated with TiO_2_ are the basis of further investigations for the use of these materials in photoactive filters, in DSSCs, or in composites with other nanoparticles acting as a catalyst substrate supporting spillover of activated hydrogen or oxygen.
